# The Role of Irisin throughout Women’s Life Span

**DOI:** 10.3390/biomedicines11123260

**Published:** 2023-12-09

**Authors:** Federica Barbagallo, Rossella Cannarella, Vincenzo Garofalo, Marta Marino, Sandro La Vignera, Rosita A. Condorelli, Lara Tiranini, Rossella E. Nappi, Aldo E. Calogero

**Affiliations:** 1 Department of Clinical and Experimental Medicine, University of Catania, 95124 Catania, Italy; federica.barbagallo@phd.unict.it (F.B.); vgarofalo985@gmail.com (V.G.); dottmartamarino@gmail.com (M.M.); sandrolavignera@unict.it (S.L.V.); rosita.condorelli@unict.it (R.A.C.); acaloger@unict.it (A.E.C.); 2 Glickman Urological and Kidney Institute, Cleveland Clinic, Cleveland, OH 44125, USA; 3 Department of Clinical, Surgical, Diagnostic and Pediatric Sciences, University of Pavia, 27100 Pavia, Italy; lara.tiranini01@universitadipavia.it (L.T.); nappi@rossellanappi.com (R.E.N.); 4 Research Center for Reproductive Medicine, Gynecological Endocrinology and Menopause, IRCCS San Matteo Foundation, 27100 Pavia, Italy

**Keywords:** irisin, bone health, metabolism, reproduction, HPG axis, polycystic ovary syndrome (PCOS), functional hypothalamic amenorrhea (FHA), gestational diabetes (GDM), menopause

## Abstract

Since its discovery, much attention has been drawn to irisin’s potential role in metabolic and reproductive diseases. This narrative review summarizes and updates the possible role played by this fascinating molecule in different physiological (puberty and menopause) and pathological (polycystic ovary syndrome (PCOS), functional hypothalamic amenorrhea (FHA), endometriosis, and gestational diabetes) conditions that can affect women throughout their entire lives. Irisin appears to be an important factor for the hypothalamic–pituitary–gonadal axis activation, and appears to play a role in the timing of puberty onset. Serum irisin levels have been proposed as a biomarker for predicting the future development of gestational diabetes (GDM). Its role in PCOS is still controversial, although an “irisin resistance” mechanism has been hypothesized. In addition to its impact on metabolism, irisin also appears to influence bone health. Irisin levels are inversely correlated with the prevalence of fractures in postmenopausal women. Similar mechanisms have also been postulated in young women with FHA. In clinical settings, further controlled, prospective and randomized clinical trials are needed to investigate the casual relationship between irisin levels and the conditions described and, in turn, to establish the role of irisin as a prognostic/diagnostic biomarker or a therapeutic target.

## 1. Introduction

Irisin is a 112 amino acid (~12 kDa) hormone, resulting from cleavage of the extracellular portion of the N-terminal membrane protein called fibronectin type III domain containing protein 5 (FNDC5). It was identified for the first time in 2012 by Boström et al. [1] in myocytes of transgenic mice undergoing intense physical activity, where this molecule appeared to be involved in the cross-talk between muscles and other tissues. This peculiarity is expressed by its name, “irisin”, referring to the Greek goddess Iris, the messenger of the Gods [2].

**Figure 1:**
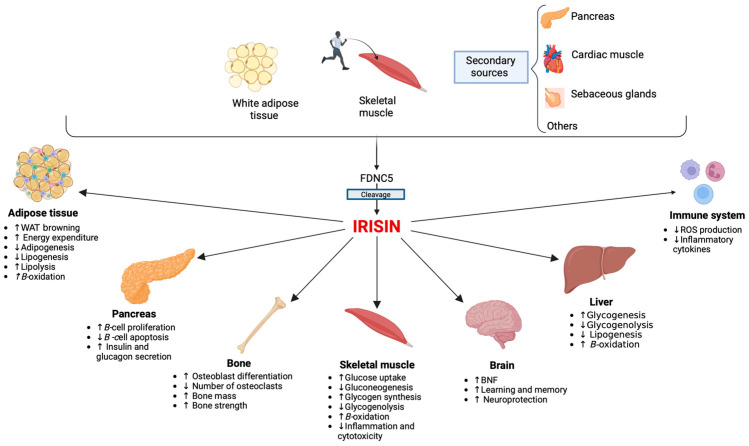
Main functions of irisin. The main sources of circulating irisin are the skeletal muscle during physical activity and white adipose tissue. Irisin has multi-spectrum functions, influencing the activity of numerous tissues and organs, mainly skeletal muscle [10], the pancreas [11], the liver [12], the brain [13], the bone [14], and the immune system [15]. Abbreviations: FDNC5, Fibronectin type III domain-containing protein 5; ROS, Reactive oxygen species; WAT, White adipose tissue; BNDF, Brain-derived neurotrophic factor.

Transgenic mice, in which irisin was first identified, were found to overexpress the *Ppargc1a* gene encoding for the peroxisome proliferator-activated receptor co-activator-1α (PGC1α). In mouse models of myocytes, irisin was able to promote the “browning processes” of white adipose tissue (WAT) and thermogenesis, mediated by uncoupling protein 1. Subsequent studies have highlighted the pleiotropic actions of irisin [3]. Among its most important effects, irisin is able to promote insulin synthesis and glucose-stimulated insulin secretion, reduce pancreatic ß-cell apoptosis in response to various harmful stimuli, and promote ß-cell proliferation [2,4,5]. Thus, irisin seems to promote the survival and restoration of the normal functional mass of ß-cells, essential for adequate glucose homeostasis [6]. Numerous studies have documented the ability of irisin to act on skeletal muscle, adipose tissue, and liver, improving insulin sensitivity, glucose uptake, reducing gluconeogenesis, glycogenolysis, and lipid accumulation, and increasing glucose storage, lipolysis, and fatty acid oxidation [7]. Therefore, irisin has been proposed as a hormone capable of increasing energy expenditure, promoting weight loss, and decreasing insulin resistance (IR) [8,9]. Irisin has become a potential target for the treatment of metabolic diseases. The main functions of irisin are shown in Figure 1.

Irisin is secreted mainly by skeletal muscle, but also by adipose tissue, the pancreas, sebaceous glands, and cardiac muscle. Irisin immunoreactivity was soon found in several other organs and tissues, including salivary glands, ovaries, testes, the rectum, intracranial arteries, the tongue, optic nerve, stomach, neuronal cells, and sweat glands [8,9] (Figure 1). For these reasons, in recent decades, irisin has been the subject of many studies investigating its possible role in the pathogenesis and development of various diseases. Numerous studies have focused on the association of irisin with metabolic diseases, suggesting its potential role as a novel target to address type 2 diabetes mellitus (T2DM) and IR. In women, it has been proposed as a new marker for gestational diabetes mellitus (GDM) [16]. Furthermore, irisin appears to be significantly related to bone health, as described in postmenopausal women [17]. Moreover, several studies have attempted to understand the role of irisin in reproductive diseases such as polycystic ovary syndrome (PCOS), although contradictory results have been reported [18].

On this basis, the present narrative review aims to summarize and update the currently existing evidence on the possible role of irisin in the main physiological and pathological conditions throughout women’s lifespan, from puberty to menopause, and to examine its involvement in the more-frequent reproductive disorders affecting women.

## 2. Irisin and Sex Differences: Towards Gender Medicine

There is currently no indicative range of values considered normal for irisin in plasma and serum. Circulating irisin levels appear to vary significantly, with concentrations reported in humans ranging from 0.01 ng/mL to 2000 ng/mL [18]. Although difference in irisin levels between women and men have been hypothesized, the results are still controversial. Previous studies have shown higher irisin levels in girls than boys [19], and in women than men [20]. Zügel et al. [21] showed that the transient increase in serum irisin after acute exercise is more pronounced in lean women than in men. In ovariectomized mouse models, the same authors found adiposity and metabolic alterations associated with increased circulating irisin and FNDC5 expression in skeletal muscle, independently of PGC-1α expression. These results suggest a compensatory mechanism, whereby higher amounts of irisin are secreted to increase energy expenditure by burning WAT or other still unknown effects [21].

Al-Daghri et al. [19], in a study conducted on 81 male and 72 female children, found that high circulating levels of irisin are correlated with impaired glucose tolerance, and that this association is more evident in girls. The authors suggest that this sexual dimorphism could be explained by hormonal differences in the two sexes. 17ß-estradiol (E_2_) could influence irisin circulation through anabolic pathways to increase muscle mass leading to the up-regulation of irisin, and promoting an irisin resistance mechanism [22]. Another explanation could concern the different distribution of brown adipose tissue (BAT) between women and men. Indeed, women have a more active BAT than men, at least in adulthood. Furthermore, the recent discovery that irisin is also secreted by WAT could partly explain the gender difference, since the distribution of WAT itself is gender dimorphic [23]. A study conducted in healthy young people showed that lean body mass was a strong positive predictor of irisin levels, with higher levels in women than men, after adjustment for lean body mass. Furthermore, the female gender was independently associated with higher irisin levels, confirming a gender dimorphism for irisin production [20].

Evidence for the sexual dimorphism of irisin distribution and effects also comes from new insights, which have identified irisin in the central nervous system (CNS). A study conducted on brain samples of marmoset and rhesus monkeys revealed the different distributions of FNDC5 and PGC1A, depending on sex. In females, the pituitary gland and posterior hypothalamus had considerably higher FNDC5 and PGC1A transcript levels than the corresponding male counterparts did. These findings demonstrate for the first time that such genes are expressed in neonatal and adult monkeys in a tissue- and sex-specific manner [24]. Further research is needed to understand whether these findings have any impact on sex-specific variations in endocrine physiology.

## 3. Irisin and Pubertal Development

Irisin appears to be an important factor activating the function of the hypothalamic-pituitary-gonadal (HPG) axis and reproductive capability [25]. Indeed, irisin and FNDC5 have been identified in the hypothalamus, pituitary gland, ovary, and testis. New insights have led to the identification of irisin in the CNS. Irisin is expressed primarily in the ventromedial nucleus and arcuate nucleus of the hypothalamus, areas known to be involved in the regulation of feeding, energy homeostasis, and reproduction [24]. Furthermore, direct contact between irisin-positive fibers and gonadotropin-releasing hormone (GnRH) neurons was identified, suggesting a direct influence of irisin on GnRH pulses. Furthermore, in mouse models, irisin administration revealed an enhancement of GnRH transcription after one hour of incubation [24]. These findings demonstrated that irisin is involved in GnRH release, and suggested that it might influence GnRH expression and, consequently, reproductive function. However, the role of irisin in the HPG axis is controversial, with opposing evidence. Ulker et al. demonstrated that daily intraperitoneal injections of irisin (100 ng/kg from postnatal day 21 for approximately 10 weeks) delayed the onset of puberty and decreased the expression of GnRH in the brain of female rats [26]. Chronic administration of irisin also affected hormone levels, by lowering serum follicle-stimulating hormone (FSH) levels and increasing serum luteinizing hormone (LH) and E_2_ levels. In addition to these changes, ovarian tissue from irisin-treated rats showed a reduction in early follicles and an increase in fibrosis. On the other hand, the timing of puberty remained unaltered by the administration of irisin to male rats. However, after prolonged exposure to irisin, male rats’ serum testosterone and LH levels, sperm count, and seminiferous tubule width were significantly increased [26]. Therefore, the authors concluded that the reproductive systems and pubertal maturation of male and female rats respond differently to irisin exposure, and long-term exposure to irisin may alter the female reproductive system. At the same time, it appears to have androgenic effects on the human HPG axis. Wahab et al. [25] suggested a role for irisin in activating the neurohormonal network that regulates the onset of puberty in mammals. Indeed, they found that FNDC5 mRNA expression in mice increases during postnatal development, and systemic irisin levels increase around puberty onset in humans. Thus, the authors suggest that an increase in FNDC5 mRNA in peripheral tissues, such as adipose tissue and muscles, and the subsequent increase in systemic irisin levels, could trigger the HPG axis activation.

The in vitro effects of GnRH on FSH and LH stimulation appear to be compromised when it is administered in combination with irisin [27,28]. The detrimental effects of irisin on GnRH-induced gonadotropin stimulation may be explained by several mechanisms. First, irisin could stimulate FSH and LH secretion directly, and it could decrease their release, exerting a negative feedback on GnRH secretion. Secondly, it can be hypothesized that the effects of irisin are comparable to those of GnRH agonists [18]. Therefore, irisin can bind to GnRH receptors on the cell surface, causing their down-regulation and increasing receptor internalization, thus making them inaccessible to GnRH. This decreases the production of gonadotropins by pituitary desensitization [18]. Consequently, irisin can stimulate FSH and LH secretion, but on the other hand, it could also compete with GnRH by reducing the release of gonadotropin. Changes in serum gonadotropin levels depend on the prevalence of one or the other effects of irisin.

According to Bastu et al. [29], the irisin precursor protein, FNDC5, can stimulate the growth of mouse ovaries by significantly increasing the number of primary and secondary follicles. Previous research had already revealed that *FNDC5* gene knockout mice had fewer antral follicles, in agreement with the findings obtained by these authors [18]. In contrast, some studies have found that irisin has detrimental effects on the ovaries and testes, significantly reducing the number of vegetative cells and Leydig cells, the sperm concentration, and sperm motility in male rats exposed to irisin [30], as well as significantly increasing ovarian fibrosis in female mice [26] and decreasing the number of primary follicles in rats. According to these studies, the physiological effects of irisin are concentration-dependent, which may suggest why the molecule has variable, or even diametrically opposite, effects on metabolism.

A network of hormones and neuroendocrine pathways, in which GnRH neurons play a major role, complexly regulates the onset of puberty. The balance between inhibitor and stimulatory signals mediated by kisspeptin/neurokinin B/dynorphin (KND), and neuropeptide Y [31] regulates GnRH neurons. The initiation of GnRH pulses confirms the onset of puberty, and allows the increased growth rate, bone maturation, epiphysis fusion, and the development of secondary sexual characteristics. Pulsatile GnRH secretion appears to be linked to body weight (fat/muscle), metabolic condition, and energy reserves during puberty [32]. Energy reserves and the metabolic state of the organism strongly influence the onset of puberty, and it is known that the hypothalamic network can only be activated when the body reaches a critical level of fat and/or muscle mass [25]. Various metabolic signals mediated by leptin, insulin, ghrelin, nesfatin, and neurokinin-β are believed to be implicated in the activation of the HPG axis [33,34]. On the other hand, increased body fat and obesity appear to be related to precocious puberty [35]. Body mass index (BMI), waist circumference, total body fat, and visceral fat tissue have all been linked to sexual maturation [36]. In terms of puberty, obese children also showed substantial variation in irisin concentration between prepubertal and pubertal children. According to previous studies, the main cause of elevated irisin levels in the obese population is puberty [37,38].

Few studies have been conducted to investigate the relationship between serum irisin levels and puberty, although IR is known to increase as a child approaches puberty. Therefore, if there is a significant connection between irisin and IR, it can be hypothesized that irisin levels also rise with entry into puberty [37].

Kutlu et al. [39] conducted a study to evaluate irisin levels in 94 girls, including 33 with central precocious puberty (CPP), 31 with precocious puberty (PP), and 30 healthy controls. The authors found that patients with CPP had higher serum irisin levels than the other groups. Furthermore, a positive statistically significant correlation has been reported between irisin levels and BMI standard deviation score (BMI-SDS), height SDS, weight SDS, bone age, uterine long axis, ovarian size, levels of baseline FSH and LH, and peak LH. Therefore, although further prospective studies are needed, the authors hypothesized that increased irisin levels could potentially be used as a marker of CPP.

Irisin levels in 20 children of similar age and pubertal stage were compared with indices of metabolic syndrome, such as impaired glucose metabolism, IR, lipid metabolism, and blood pressure [38]. The results showed that obese children with impaired glucose tolerance had higher irisin levels, followed by obese children with normal glucose tolerance, while the lowest levels were found in normal-weight children. Baseline irisin was strongly correlated with pubertal stage, high-density lipoprotein cholesterol (HDL-c), and a homeostasis model of assessment in a multiple linear regression analysis (HOMA-R), but not with age, gender, BMI, or any other metabolic syndrome parameter. Furthermore, prepubertal children had significantly lower irisin concentrations than pubertal youngsters. Changes in irisin were not substantially correlated with changes in BMI or with any other parameter in longitudinal studies, but they were strongly correlated with the onset of puberty, changes in fasting blood glucose, and with changes in 2 h blood glucose in an oral glucose-tolerance test. Therefore, changes in glucose metabolism characteristics were substantially related to changes in irisin levels. On the other hand, the reported increase in irisin has been suggested to influence the onset of puberty. These debated findings imply that factors other than glucose metabolism also have a major impact on irisin levels.

In conclusion, animal and human studies have shown controversial evidence regarding the role of irisin in puberty. A different response of the reproductive system to irisin exposure has been reported in mice [26]. The timing of puberty was delayed after irisin administration in female rats, whereas it remained unaltered in male rats [26]. In humans, girls with CPP demonstrated higher serum irisin levels than controls, suggesting a potential role of irisin as a marker of CPP. However, when evaluating both male and female children, irisin levels were not related to sex [38]. Further studies on animals and humans are needed to better clarify the role of irisin in the male and female organism.

## 4. Irisin and Polycystic Ovarian Syndrome (PCOS)

Polycystic ovarian syndrome (PCOS) is the most common endocrinopathy that affects women of reproductive age. It has a prevalence ranging from between 5 and 20%, depending on the different diagnostic criteria used and the population studied [40].

The pathogenesis of the disease is not completely clear, and the clinical manifestations are extremely variable. According to the Rotterdam criteria, at least two of the following three criteria are required to diagnose PCOS: (1) menstrual disorders (oligo-amenorrhea and/or anovulation); (2) clinical or biochemical hyperandrogenism; and (3) polycystic ovarian morphology (PCOM) [41]. Although these diagnostic criteria do not consider the dysmetabolic background of PCOS, it is widely recognized that patients with PCOS commonly have metabolic disorders. The prevalence of obesity is higher in patients with PCOS (relative risk (RR): 2.77; 95% CI: 1.88 to 4.1), compared to those without PCOS [42]. Furthermore, up to 80% of PCOS women exhibit IR, which is independent of BMI and plays an important role in the clinical presentation and development of several metabolic alterations [43]. Genome-wide association studies of single-nucleotide polymorphisms showed that granulosa cells of PCOS patients expressed genes linked to insulin resistance, including protein kinase AMP-activated catalytic subunit alpha 2, matrix metallopeptidase 9, and haptoglobin [44]. As reported above, many studies have shown that irisin improves IR through several mechanisms, including increasing insulin receptor sensitization in skeletal muscle and heart, improving hepatic glucose and lipid metabolism and the functions of pancreatic β cells, and promoting the transformation of WAT into BAT [45]. Therefore, many studies have been conducted to better understand the relationship between irisin and insulin. A negative correlation between irisin and insulin levels has been previously reported in healthy people [46]. However, as insulin sensitivity decreases, the relationship between irisin and insulin becomes positive.

The role of irisin in the development of PCOS is controversial. Serum irisin levels in patients with PCOS, first reported by Chang et al., were found to be higher in PCOS compared to controls, suggesting a possible role in the development of the disease [47]. On the other hand, other studies have not reported a significant difference between PCOS and healthy women with respect to irisin levels [48], and even lower irisin levels have been found in women with PCOS [49].

**Figure 2:**
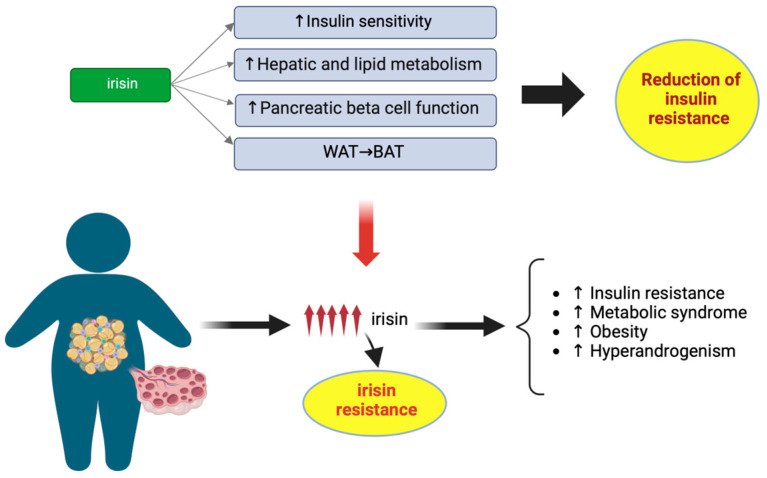
Schematic representation of the irisin-resistance hypothesis in women with polycystic ovarian syndrome (PCOS). The role of irisin in PCOS is controversial. Irisin can improve insulin resistance through several mechanisms, in healthy people. Increased irisin secretion in PCOS patients may be a protective mechanism, to compensate for reduced insulin sensibility and other metabolic conditions typical of these patients. However, the excessive increase in irisin can also induce an irisin resistance, similar to the well-known phenomenon of leptin resistance, which in turn leads to a greater risk of the metabolic consequences of PCOS. Abbreviations: WAT, White adipose tissue; BAT, Brown adipose tissue.

A recent meta-analysis including eight studies, with 918 PCOS patients and 529 controls, demonstrated that irisin levels were at least 45.78 ng/mL (95% confidence interval CI) (12.45, 79.12, *p* = 0.007) higher in PCOS patients, compared to healthy controls [50]. However, another meta-analysis including the same studies showed that, adjusting for BMI, serum irisin levels in PCOS patients were similar to those of healthy controls [51]. Irisin levels were higher in PCOS patients with higher BMI than in those with lower BMI (d = 0.36, 95% CI 0.15, 6 to 0.56) [51]. Therefore, circulating irisin levels appear to be influenced by BMI [50]. In both PCOS and non-PCOS women, irisin levels were higher in overweight and obese women than in those of normal weight [52,53]. Weight loss led to a significant reduction in irisin levels (15%), while weight regain brought irisin levels back to baseline levels [53]. In the meta-analysis conducted by Cai et al., it was also reported that irisin decreased 2 h later in response to euglycemic hyperinsulinemia in PCOS patients, and to a greater extent than in healthy controls (d = −0.32, 95% CI −0.53 to −0.11) [51]. These results may imply the possible role of irisin against IR, or irisin resistance in women with PCOS. Irisin levels have been associated with metabolic syndrome and its components in PCOS patients [54]. Therefore, it has been hypothesized that increased irisin secretion in PCOS patients may be a protective mechanism to compensate for decreased insulin sensibility and other metabolic conditions. As a biomarker, increased irisin levels may occur before the development of metabolic consequences in PCOS patients. On the other hand, it could also suggest IR, similar to the well-known phenomenon of leptin resistance (Figure 2).

Given the significant clinical heterogeneity, several classifications have been proposed for distinguishing the different phenotypes of PCOS. A recent study evaluating differences in irisin levels in PCOS women, based on various phenotypes, reported that serum irisin levels were associated with hyperandrogenism, but not with oligo-anovulation or PCOM presence [55]. Li et al. demonstrated that elevated irisin levels in PCOS women were associated with androgen excess, assessed through the free androgen index [56]. Hyperandrogenism is also an important inducer of IR, which highlights the need to further investigate the relationship between irisin, hyperandrogenism, and IR. Furthermore, previous studies have also shown that androgen levels are inversely correlated with BAT thermogenesis, and that PCOS women have reduced BAT levels [57,58]. Therefore, an increase in, and activation of, BAT could be considered a possible treatment strategy for PCOS and its metabolic consequences. A recent study showed that irisin can improve body weight, thermogenesis, and insulin sensitivity of dehydroepiandrosterone-induced PCOS mice, by activating BAT function. Moreover, irisin treatment improved the estrous cycle, reduced the levels of testosterone, the anti-Müllerian hormone, LH, and LH/FSH ratio, and decreased the formation of ovarian cystic follicles in PCOS mice [59]. Therefore, it has been proposed that irisin plays a role not only in the pathogenesis of the disease, but also as a possible therapeutic option. A recent meta-analysis reported a reduction in irisin levels after metformin treatment in PCOS patients [60]. However, this meta-analysis included only four studies, with significant heterogeneity in terms of sample size, dosage, and treatment duration. The reduction in irisin levels may be related to the significant reduction in insulin and IR after metformin treatment. Previous studies have reported that metformin can up-regulate irisin expression through AMP-activated protein kinase/sirtuin 1/peroxisome proliferator-activated receptor gamma coactivators-1α (AMPK/SIRT1/PGC-1α) signaling pathway [52].

In conclusion, currently, contradictory results link irisin to PCOS. Irisin levels in PCOS patients were found to be higher [47], similar [48], or even lower [49], compared to controls. These differences may be related to a significant heterogeneity of published studies. Irisin levels can be influenced by several factors, particularly BMI. They can also relate to different stages of the diseases. Indeed, irisin could increase in the first phase as a protective mechanism to compensate for reduced insulin sensibility in PCOS patients. However, over time, the excessive increase in irisin can also cause irisin resistance, which in turn leads to a greater risk of metabolic consequences of PCOS. The findings summarized in this paragraph suggest a possible role for irisin in the etiology of PCOS, in the development of metabolic comorbidities, and as a possible new therapeutic strategy, which deserves to be further clarified.

## 5. Irisin and Functional Hypothalamic Amenorrhea

Functional hypothalamic amenorrhea (FHA) is a form of chronic anovulation not related to organic causes, but resulting from various stressors. It is generally associated with three main causes: weight loss, excessive physical exercise, and psychological stress, which can occur alone or, more often, in combination [61]. FHA is one of the most common reproductive disorders in women of childbearing age, and is responsible for 20–35% of secondary cases of amenorrhea and for approximately 3% of cases of women with primary amenorrhea [62].

FHA is characterized by the suppression of GnRH pulsatility, with the consequent reduction in LH and FSH secretion, and, therefore, hypogonadotropic hypogonadism. Although there is no underlying organic cause, some cases of FHA can take a long time to recover, and can lead to major complications, primarily in bone health, due to estrogen deficiency [63]. In young women, estrogens are the most important hormones that regulate bone metabolism, inhibit bone remodeling, decrease bone resorption, and maintain bone formation [63]. Current guidelines suggest performing baseline dual-energy X-ray absorptiometry (DXA) to evaluate bone density of the spine and hip in adolescents or women with FHA after six months of amenorrhea, or even earlier in those patients with a history of these conditions, or with severe nutritional deficiency and/or skeletal fragility [61].

In addition to estrogen deficiency, irisin depletion may also contribute to the deterioration in bone health seen in these patients. Indeed, previous studies have reported the potential role of irisin on bone metabolism, through both direct and indirect effects. In mice, irisin enhances osteoblast activation and inhibition of NF-κB ligand activating receptor (RANKL)-mediated osteoclastogenesis and, in turn, increases trabecular and cortical thickness and trabecular density [14]. This mechanism has also been confirmed in humans [64]. The effects of irisin on bone metabolism could also depend on BAT. Indeed, the adipogenesis of this tissue appears to be physiologically related to bone health, and its defective brown adipogenesis has been correlated with bone loss [65]. Irisin levels have also been negative correlated with serum sclerostin, an inhibitor of osteoblast differentiation and bone formation [66]. The in vivo and in vitro studies that have investigated the relationship between irisin and bone metabolism will be discussed later (see paragraph 6 for review). Indeed, previous studies have shown that serum irisin levels are inversely correlated with the prevalence of fractures in postmenopausal women [17]. Therefore, irisin has been proposed as a potential marker for monitoring osteoporosis. As in postmenopausal women, a potential role for irisin in the bone health of young women with FHA could be hypothesized.

On the other hand, it has recently been shown that physical activity increases irisin expression in skeletal muscle [1]. Consequently, it must be taken into account that some patients with FHA are athletes who take part in intense physical exercise. For this reason, the evaluation of the levels and potential role of irisin on bone metabolism in patients with FHA should consider the underlying causes (intense physical activity, malnutrition, or psychological stress), although this is complicated, due to the frequent overlap of these three factors.

To our knowledge, only two studies investigated serum irisin levels in patients with FHA. In 2014, Singhal et al. enrolled 85 women (38 amenorrheic athletes, 24 eumenorrheic athletes, and 23 non-athletes), aged 14 to 21 years, to study the relationship between irisin and bone health. Across all groups, irisin levels were positively associated with spine, femoral neck, and whole-body bone mineral density (BMD) Z scores. Irisin was also associated with total and trabecular volumetric BMD and estimates of bone strength, including stiffness and failure load. The authors found lower irisin levels in athletes with amenorrhea compared to athletes without amenorrhea and non-athletes, and these differences persisted, even after correcting the data for fat and lean mass [67]. They speculated that decreased irisin levels in amenorrheic athletes could represent a defensive mechanism to preserve energy by reducing BAT adipogenesis. In support of this hypothesis, lower levels of irisin [68] and lower presence of BAT have been found in conditions of extreme malnutrition, such as anorexia nervosa [69].

Another study showed lower irisin levels in non-athletes and non-anorexic women with FHA, compared to healthy controls [70]. The authors considered physical exercise and eating disorders as confounding factors because they influence serum irisin levels independently, and decided to focus on patients with FHA due to psychogenic stress. However, BMI was statistically significantly reduced in FHA patients compared to controls (19.4 ± 2.3 vs. 22.7 ± 0.7 kg/m^2^, *p* < 0.001). The FHA group showed lower irisin levels associated with significantly reduced BMD parameters that did not reach the severity of osteopenia. Therefore, the authors hypothesized that irisin could predict DXA outcomes and, in turn, irisin measurement could allow earlier targeted therapies, to avoid osteoporosis [70].

In conclusion, the findings discussed in this section do not allow a univocal conclusion, due to the small number of studies investigating the relationship between irisin and FHA. However, given the growing number of young women with FHA, further studies are needed to evaluate the possible role of irisin as a predictor of bone deprivation, and as an indirect indicator of nutritional status in these patients.

## 6. Irisin and Endometriosis

Previous preclinical and clinical studies have suggested that irisin has anti-inflammatory properties [71]. Mechanisms involved in the anti-inflammatory functions of irisin include reducing the production of pro-inflammatory cytokines (such as interleukin-6 (IL-6) and tumor necrosis factor-α), increasing the production of anti-inflammatory cytokines, reducing macrophage proliferation, inducing polarization of alternative-type macrophages, inhibiting pathways of increased vascular permeability, and preventing the formation of inflammasomes (such as toll-like receptor 4/myeloid differentiation factor 88 downstream pathways) [15,72].

It is now known that elevated levels of various pro-inflammatory cytokines play an important role in the pathogenesis of endometriosis. Endometriosis is a common inflammatory disease, characterized by the presence of tissue outside the uterus, which resembles the endometrium, primarily on pelvic organs and tissues. It affects approximately 5–10% of women of reproductive age, and reduces women’s quality of life, due to dysmenorrhea, chronic pelvic pain, irregular uterine bleeding, and infertility [73]. To our knowledge, only one study has been conducted to investigate irisin levels in patients with endometriosis [74]. Kaya Sezginer et al. [74] found that serum irisin levels were increased in endometriosis patients compared to controls, which appeared to correlate with BMI and C-reactive protein. These findings contrast with other studies, which indicated lower serum irisin levels compared to the control in inflammation-related diseases [71,75]. The authors speculated that the increase in irisin might be an adaptive response to compensate for the increased inflammation in endometriosis [74].

Kuloglu et al. [76] performed a study on samples of human ovarian cancer, breast cancer, and cervix cancer that were immunohistochemically stained with anti-irisin antibodies. The authors demonstrated that ovarian endometriosis and mucinous carcinomas have much higher irisin immunoreactivity than benign tumors. Increased irisin immunoreactivity in breast, ovarian, cervical and endometrial tumors suggests a crucial function for this peptide in cancer development.

Finally, to date, only one study has reported an increase in irisin levels in patients with endometriosis, perhaps as an adaptive response to compensate for the increased inflammation underlying this disease. Further studies are needed to investigate the possible anti-inflammatory role of irisin in patients with endometriosis.

## 7. Irisin and Gestational Diabetes Mellitus

GDM is a condition that affects pregnant women, and is characterized by hyperglycemia during pregnancy. According to the American Diabetes Association (ADA), GDM is defined as the onset of impaired glucose tolerance during the second or third trimester of pregnancy [77]. According to the data collected and analyzed by the International Diabetes Federation (IDF), the global prevalence of GDM is approximately 14%, although it varies depending on the population studied [78].

Pregnancy is characterized by significant changes in the mother’s metabolic processes, thus facilitating the provision of adequate energy and nutrients to the developing fetus. As a result, pregnant women experience a period of reduced insulin sensitivity during mid-gestation, which intensifies during the third trimester. This subsequently leads to reduced glucose uptake by maternal tissues and increased glucose production through gluconeogenesis [79]. However, in a significant percentage of pregnancies, the IR state is exacerbated, resulting in adverse maternal metabolic conditions and abnormal fetal growth [80]. The mechanisms underlying IR during pregnancy include increased adiposity and the action of placental hormones with diabetogenic effects, such as estrogen, progesterone, cortisol, human chorionic gonadotropin, and prolactin [81,82,83].

GDM has a multifactorial pathogenesis, including both genetic and environmental factors, but the exact mechanism is not fully understood [16]. The presence of GDM during pregnancy increases the risk of perinatal morbidity, pre-eclampsia, fetal macrosomia, and shoulder dystocia, and increases the risk of developing T2DM, cardiovascular disease, and obesity, in both mother and offspring [77]. Early diagnosis and appropriate management of GDM can help mitigate adverse effects in both the mother and fetus, as well as protecting them from potential long-term complications. Consequently, studies have been undertaken to investigate the association between irisin and metabolic disorders, since irisin has a significant impact on metabolism. Recent research has shed light on the potential role of irisin as a possible biomarker for the early diagnosis of GDM.

The concentrations of irisin increase in a statistically significant manner during pregnancy and particularly in the second and third trimesters, compared to the early stages of gestation. Furthermore, serum irisin levels are significantly higher in pregnant women than in non-pregnant women [84]. Maternal irisin levels are negatively correlated with the HOMA-IR in most studies [85,86], although some of them have not found any correlation [87] or even a positive correlation [88,89]. However, it has been hard for studies to evaluate the association between irisin levels and maternal HOMA-IR, considering potential confounding variables such as age, BMI, physical activity, and nutritional status, which influence circulating irisin levels [23]. To reduce the potential influence of confounding factors, Cai et al. used multiple linear regression analysis, and found an inverse association between maternal serum irisin levels and HOMA-IR [90]. This suggests that the increase in irisin during pregnancy is an adaptive response, to compensate for the increase in IR. Furthermore, maternal irisin showed a negative association with fasting plasma glucose (FPG) [90]. Several possible interpretations have been proposed to explain this finding. Experimental evidence has shown that elevated serum irisin levels within one hour can significantly increase intracellular glucose, through increased expression of glucose transporter type 4 (GLUT4) in skeletal muscle [91]. Second, irisin can inhibit hepatic gluconeogenesis by decreasing the expression of glucose-6-phosphatase and phosphoenolpyruvate carboxykinase in the liver [92]. Overall, maternal serum irisin may influence FPG by improving glucose uptake and inhibiting gluconeogenesis. However, a non-significant inverse association was found between maternal irisin and 1 and 2 h glucose levels during the oral glucose tolerance test (OGTT). It is plausible that postprandial glucose levels, unlike FPG, are more likely to be influenced by external variables such as dietary carbohydrate intake and gastrointestinal absorption functions [90].

In recent years, several studies have investigated the relationship between serum irisin levels and the development of GDM. Cui et al. conducted a systematic review and meta-analysis to comprehensively discuss the role of irisin in the onset and development of GDM. Data analysis showed that serum irisin levels were significantly lower in the GDM group than in the control group during pregnancy, suggesting that irisin may play an important role in the onset and development of GDM [93]. The results of this study are consistent with two other previously published systematic reviews and meta-analyses [94,95]. A recent study demonstrated a significant reduction in serum irisin levels in pregnant women with GDM, compared to healthy women at 24–28-week gestation. Furthermore, fasting and post-OGTT glucose, insulin levels, and HOMA-IR were significantly higher in women with GDM [16]. These findings are in line with the available research. Yuksel et al. also observed a decrease in serum irisin in women with GDM [86], and Kuzmicki et al. found a significant increase in serum irisin levels in pregnant women compared to non-pregnant women. This increase was much less pronounced in patients with GDM [85]. Another study found a negative association between irisin levels and pre-pregnancy BMI, HOMA-IR, HbA1c, and fasting glucose and insulin levels. In addition, pregnant women with IR had lower serum irisin levels, regardless of GDM status [96]. Other data reported a negative correlation between serum irisin and BMI and glucose 2 h after a meal, while it showed a positive correlation with total cholesterol, triglycerides, and LDL cholesterol levels [97].

Serum irisin levels have been proposed as a biomarker to predict the future development of GDM. Wang et al. measured maternal irisin levels during the first trimester of pregnancy, and found significantly lower levels in women who subsequently developed GDM compared to those who did not, after adjusting for confounding factors (BMI, insulin, FPG, and lipid metabolism) [98]. In a similar study, irisin levels during the first trimester of pregnancy were reduced in pregnant women subsequently diagnosed with GDM, and in healthy controls they were positively correlated with fasting insulin levels and IR, measured by HOMA-IR [87]. As mentioned above, irisin improves IR during pregnancy. Low levels during the first trimester could be associated with a pre-existing metabolic condition, leading to inadequate compensation by pancreatic ß-cells and insulin secretion, with increased susceptibility to GDM [87]. Another study investigated the diagnostic value of first-trimester adipokines and placental markers in predicting macrosomia. Mothers of macrosomic infants had higher levels of placental growth factor and irisin and lower levels of fetuin-A than mothers of normal-weight infants. Neonatal weight correlated positively with maternal irisin, and negatively with fetuin-A concentrations. Multiple regression analysis showed that only serum irisin levels were a significant predictor of birth weight. These results suggest that maternal irisin may be an early biomarker of macrosomia [99].

Other studies have looked at irisin levels in cord blood. However, currently, the available evidence suggests that levels of this hormone in maternal serum do not appear to correlate with levels in cord blood, either in women with or without GDM [93,100]. This may be because the main source of fetal irisin is the placenta, fetal muscle tissue, and fetal adipose tissue, which is browner and contributes to irisin production [100]. However, a study conducted in China suggests that babies with cord-blood irisin levels above a certain threshold have a significantly increased risk of macrosomia and a high birth-weight index. There is also a non-linear relationship between irisin levels and fetal macrosomia, suggesting that higher irisin levels increase the risk of macrosomia. However, the sample size was too small, and further confirmation is needed [59].

Regarding breast milk, low levels of irisin have been found in women with GDM, not only in serum, but also in colostrum and mature breast milk. Continued feeding of breast milk with reduced irisin levels may have negative effects on infant weight and lipid regulation. The relationship between low irisin levels in breast milk and newborn weight requires further studies, to be clarified [101].

In addition to being a biomarker for GDM, irisin may represent a new target for placental function. Drewlo et al. demonstrated that irisin promotes differentiation and improves trophoblast function in the human placenta, which is impaired in cases of abnormal placentation. Indeed, irisin induces trophoblast cell–cell fusion and growth in placental explants, suggesting a role in promoting trophoblast differentiation. This is due to irisin’s activation of the AMPK signaling pathway in trophoblast cell lines, suggesting a potential role for irisin as a marker of placental function [102].

The findings summarized in this paragraph suggest the potential role of irisin as a biomarker for predicting the future development of GDM, which represents a significant health risk for both pregnant women and their unborn children. While much is still unknown about the precise mechanisms underlying this condition, emerging research on irisin offers hope for better prevention and management strategies. The potential link between irisin and GDM highlights the importance of a healthy lifestyle during pregnancy, including regular physical activity and a balanced diet. By promoting the release of irisin, pregnant women may be able to improve their insulin sensitivity. Irisin could prove to be a valuable tool in the fight against GDM, improving health outcomes for both mothers and their babies.

## 8. Irisin and Menopause

Menopause is a natural biological event that marks the end of a woman’s reproductive age, and typically occurs between her late forties and early fifties. Menopause is associated with a decline in BMD, resulting in an increased risk of osteoporosis and fractures [103]. Irisin has been identified as a potential factor associated with osteoporosis, a condition that causes a significant socioeconomic burden worldwide, particularly in postmenopausal women [17,104].

A systematic review/meta-analysis of seven studies, involving 1018 participants, found that serum irisin levels were significantly lower in middle-aged and older adults with osteoporosis, compared to those without osteoporosis. The analysis also showed that irisin levels were even lower in postmenopausal women and those with a history of bone fractures. Furthermore, a weak positive correlation was found between irisin levels and BMD at the femoral neck or lumbar spine. These findings suggest that postmenopausal women with osteoporosis may have significantly lower irisin levels, suggesting a potential link between irisin and the development of osteoporosis in this population [17].

**Figure 3:**
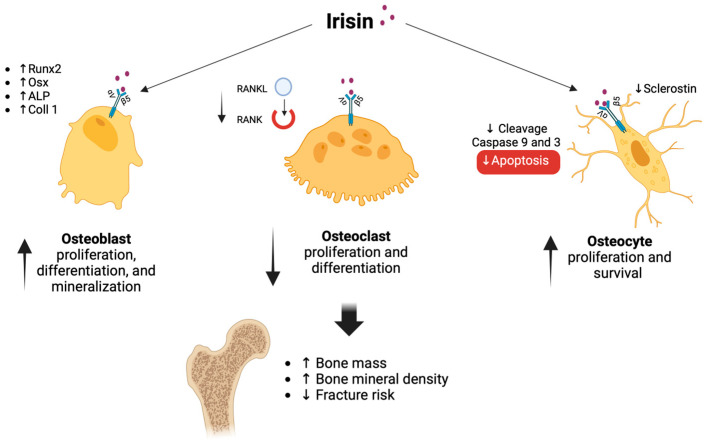
Schematic representation of the action of irisin on bone cells. Irisin acts on osteoblasts, osteoclasts, and osteocytes, through αV/β5 integrin receptors. Irisin increases the expression of osteoblast transcriptional regulators (Runx2, Osx) and early marker genes of osteoblast differentiation (ALP, ColIa1), resulting in increased osteoblast proliferation, differentiation, and mineralization. Irisin treatment inhibits the RANKL receptor in osteoclasts, leading to a decrease in their proliferation and differentiation. In osteocytes, irisin decreases the expression of sclerostin and inhibits caspases 9 and 3 cleavage, reducing apoptosis and promoting osteocyte proliferation and survival. Elevated irisin levels are positively correlated with bone mass and bone mineral density, while negative correlated with osteoporotic fracture risk. Abbreviations: Runx2, Runt-related transcription factor 2; Osx, Osteoblast-specific transcription factor osterix; ALP, Alkaline phosphatase; ColIa1, Collagen type 1 alpha 1 gene; RANK, Receptor activator of nuclear factor-kappa B; RANKL, Receptor activator of nuclear factor-kappa B ligand.

In in vitro studies, irisin is associated with an increase in bone formation and a decrease in bone resorption, resulting in a reduced risk of osteoporosis in postmenopausal women. At the bone tissue level, irisin acts on target cells, through αV/β5 integrin receptors. This influences the proliferation, differentiation and activity of osteoblasts, osteoclasts, and osteocytes [105]. Irisin treatment of pre-differentiated osteoblasts increases the phosphorylation of Extracellular signal-Regulated Kinase and p38, resulting in higher mRNA levels of osteoblast transcriptional regulators (Runt-related transcription factor 2 (Runx2), Osteoblast-specific Transcription Factor Osterix (Osx)) and early osteoblast differentiation marker genes (alkaline phosphatase (ALP), collagen type 1 alpha 1 gene (ColIa1)), which lead to increased proliferation, differentiation, and mineralization of osteoblasts [106]. Furthermore, by activating the Wnt signaling pathway, irisin increases the levels of ß-catenin protein, which translocates into the nucleus and increases the expression of RUNX, thus improving the proliferation and differentiation of osteoblasts [107]. Irisin treatment inhibits the RANKL receptor in osteoclasts, leading to a decrease in their proliferation and differentiation [108]. Moreover, irisin reduces the expression of specific osteoclast genes, by inhibiting the expression of NF-κB [64]. Irisin treatment also showed effects on osteocytes. Binding to αV/β5 receptors activates the focal adhesion kinase pathway, leading to activation of β-catenin, increased activating of transcription factor 4, and decreased sclerostin expression. This leads to increased bone formation. Also, in osteocytes, irisin inhibits caspase 9 and 3 cleavage, by reducing apoptosis and promoting osteocyte proliferation and survival [109,110]. Contrary to previous claims, in vivo studies reported that irisin treatment increased serum sclerostin levels in a dose-dependent manner when injected continuously, once daily, for 6 days [111]. These differences could be explained by the parathyroid paradigm, which exerts anabolic and catabolic effects on the skeleton, depending on whether it is administered intermittently or continuously [112]. Therefore, intermittent irisin secretion, such as occurs with physical activity, could promote bone remodeling, while chronic irisin administration could result in bone catabolism, by increasing sclerostin levels [113]. Figure 3 illustrates the main effects of irisin on bone cells.

In vivo studies have shown that irisin administration increases trabecular- and cortical-bone thickness, volume, and mass. Furthermore, a positive association was observed between serum irisin levels and BMD, and a negative association was found between osteoporosis, ovariectomy, and inflammatory bone disease [104]. Morgan and coworkers investigated the effect of irisin in preventing osteoporosis after ovariectomy in rats. They measured levels of bone biomarkers such as bone alkaline phosphatase (BALP), tartrate-resistant acid phosphatase (TRAP), and osteocalcin. BALP is released by osteoblasts and is a marker of bone mineralization. TRAP, on the other hand, is produced by osteoclasts and is a marker of bone resorption. Finally, osteocalcin reflects osteoblast activity. In the irisin-treated group, the average levels of bone biomarkers, calcium, and phosphorus showed a significant decrease, compared to the untreated group. This demonstrates that irisin treatment results in the return of serum biomarkers of bone turnover in serum to near-normal levels, compared to untreated ovariectomized mice. Moreover, histological examination showed that irisin treatment preserved the regular bone architecture [114]. The estrogen deficiency that occurs during menopause accelerates bone aging. The absence of estrogens triggers the production of the receptor for RANKL, which acts as the main catalyst for osteoclastogenesis and as a suppressor of osteoprotegerin, a receptor that retains RANKL. This, in turn, increases the bioactivity of RANKL, and subsequently leads to bone resorption, resulting in bone loss [115]. In a study conducted on osteoporotic postmenopausal women, serum irisin levels were reduced, suggesting its potential role in the pathogenesis of osteoporosis [116]. Palermo et al. studied the relationship between irisin and body composition in postmenopausal women with osteoporosis and the impact of irisin levels on vertebral fragility fractures, and evaluated the effect of daily physical activity on irisin levels, using a wearable metabolic Holter, in postmenopausal women with fragility fractures. Their data confirm an inverse association between vertebral fragility fractures and serum irisin levels, but no association was found with lean mass or BMD. Their results also highlight the role of this myokine, which may play a more relevant role in bone quality rather than bone mass, and may positively influence bone strength. Furthermore, the level of daily physical activity appears to have little effect on irisin levels [117]. A more recent study investigated the role of serum irisin levels in postmenopausal women in predicting the risk of hip fractures. Total body BMD and total hip BMD were positively correlated with irisin levels, which were lower in women with hip fractures compared to normal control women [118]. These findings are consistent with those of Yan et al., who also found that irisin levels were negatively correlated with age and time since menopause, and positively with BMD of the femoral neck and lumbar spine [119]. The positive correlation between irisin and BMD may be due to advanced age and significant muscle loss associated with menopause. Indeed, a positive correlation was found between serum irisin and quadriceps cross-sectional area, showing that serum irisin levels were significantly lower in patients with sarcopenia [120]. In postmenopausal women with primary hyperparathyroidism, irisin levels are lower than in controls [121]. This is supported by the results of other studies showing that irisin levels are inversely correlated with parathyroid hormone (PTH) in postmenopausal women with low bone mass [122]. Irisin treatment also appears to reduce PTH receptor expression in osteoblasts, suggesting an anabolic effect on bone, not only by stimulating osteoblast formation and function, but also by reducing the effect of PTH on these cells [121].

Decreased estrogen levels in the menopausal years are associated with an increased risk of cardiovascular disease and stroke [123]. Irisin has been found in cerebrospinal fluid and various brain regions, suggesting that it may cross the blood–brain barrier, or is produced at this level [89]. Irisin-specific receptor-mediated signaling pathways are present in various cells, including neurons in the brain [124]. Irisin has been shown to increase cell proliferation and improve synaptic plasticity and memory function in rodents [125]. Furthermore, irisin treatment has demonstrated neuroprotective effects, reduced infarct volume, and improved neurological function in mouse models of stroke [126]. These data suggest the potential role of irisin in brain metabolism and its potential use as a post-stroke rehabilitation therapy [127].

Regarding physical activity in postmenopausal women, Kim et al. reported increased irisin levels after moderate-intensity endurance exercise such as an uphill walking, also with an improved lipid profile and increased brown fat, protecting postmenopausal women from cardiovascular and metabolic risk [128]. Increased irisin levels upregulate lipolysis-related genes such as *hormone-sensitive lipase*, *adipose triglyceride lipase*, and *fatty acid binding protein 4*, reducing lipid accumulation in adipocytes and increasing glycerol release. Furthermore, increased expression of GLUT4 in brown-fat cells facilitates glucose uptake [129].

Recently, whole-body cryotherapy (WBC) has been shown to reduce abdominal obesity in postmenopausal women with metabolic syndrome. Indeed, exposure to WBC causes an increase in serum concentrations of irisin and IL-6, suggesting a possible role in the reduction of abdominal obesity and as a therapeutic option in these patients [130]. Cryotherapy stimulates the sympathetic nervous system, causing vasoconstriction of the dermis and subcutaneous tissue. After the procedure, vasodilation and contraction of muscle cells occur, inducing the *FNDC5* gene expression through increased levels of peroxisome proliferator gamma coactivator 1-α (PGC-α1). This translates into the release of irisin and consequent increases in the levels of the uncoupling protein-1, which in turn causes the browning of WAT and mediates proton transport across the inner mitochondrial membrane, bypassing ATP synthase. Therefore, energy is dissipated in the form of heat, leading to the rapid depletion of cellular storage substrates, which is associated with weight loss [130]. Similar results were obtained in a study on ovariectomized obese rats. Administration of the isoflavone phytoestrogen genistein (GEN) increases serum irisin levels. This is because GEN leads to increased expression of PGC-1α, which regulates irisin production and consequently the darkening of the adipose tissue and an increased adaptive thermogenesis [131].

There are currently no studies of irisin levels in postmenopausal women before and after hormone replacement therapy (HRT), and further studies are needed. However, a study in men with late-onset hypogonadism (LOH) and metabolic syndrome reported a positive association between serum irisin and testosterone levels, and HRT induced a statistically significant increase in irisin levels in these patients. These results suggest a potential association between irisin and testosterone treatment in male patients with LOH and metabolic syndrome, and shed light on the role of irisin as a mediator of the effects of physical activity on adipose tissue and metabolism [132].

Although the role of irisin on bone health is less clear compared to the metabolic effects of this molecule, the findings described in this paragraph demonstrated the positive effect of irisin on bone health, suggesting a possible role for this hormone as a marker of osteoporosis in postmenopausal women.

## 9. Conclusions

**Figure 4:**
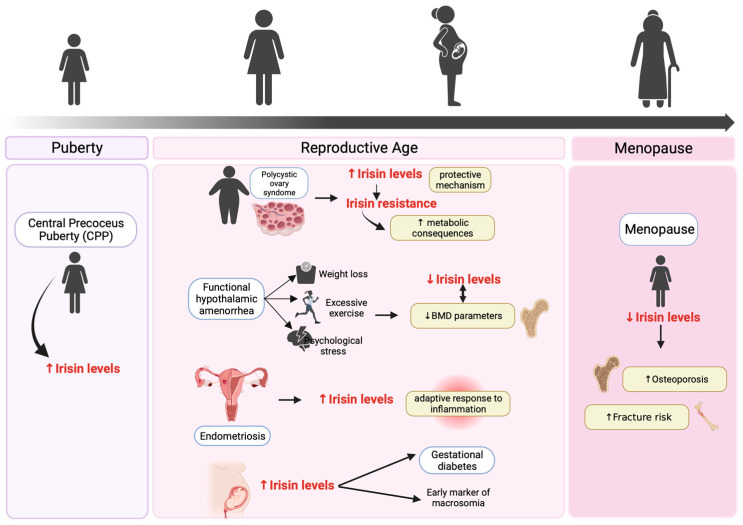
Role of irisin throughout a woman’s life. The figure summarizes the role of irisin in the main physiological and pathological conditions of a woman’s lifespan, from puberty to menopause, and its involvement in the most frequent reproductive disorders that affect women. Abbreviations: BMD, bone mineral density.

Since its discovery, the potential role of irisin on metabolic and gonadal diseases has attracted much attention. This review summarizes the possible role of this myokine in different physiological (puberty and menopause) and pathological (PCOS, FHA, endometriosis, GDM) conditions, which can affect women throughout their lives (Figure 4). Irisin appears to be an important trigger for HPG axis activation, and may play a role in the timing of puberty onset. Increased serum irisin levels have been reported in children with CPP, suggesting irisin as a potential diagnostic marker for this condition.

The positive effects in the regulation of hepatic and pancreatic functions, adipose tissue, and energy expenditure, have made irisin a possible new therapeutic target for the treatment of metabolic diseases. Irisin levels were found to be significantly lower during the first trimester of pregnancy in women who subsequently developed GDM, compared to those who did not develop it in the subsequent months. Therefore, serum irisin levels have been proposed as a biomarker to predict the future development of GDM. Interestingly, irisin could be a marker not only for GDM, but also for macrosomia and placental function. Despite the misnomer of PCOS, this syndrome is not a problem of ovarian cysts, but it is a truly complex disorder, with important metabolic consequences. An “irisin resistance” mechanism, similar to the well-known phenomenon of leptin resistance or IR, has been hypothesized in patients with PCOS. Although the results are still controversial, irisin appears to be involved in the etiology, the development of metabolic comorbidities, and even the treatment of this complex disease.

In contrast to its metabolic effect, the influence of irisin on bone health is still a matter of debate. Preclinical studies have shown that irisin can influence bone metabolism, through both direct and indirect mechanisms. In postmenopausal women, irisin levels are inversely correlated with bone fracture frequency. Similar mechanisms have also been hypothesized in young women with FHA.

Finally, previous preclinical and clinical studies have shown the anti-inflammatory effect of irisin. To date, only one study has reported increased irisin levels in patients with endometriosis, perhaps as an adaptive response to compensate for the increased inflammation underlying this disease.

It is important to keep in mind that, apart from menopause and GDM, where the results seem to support a univocal direction, the findings regarding the role of irisin in the other conditions described in this review are often controversial (puberty and PCOS) or based on too few studies (endometriosis and FHA).

Irisin offers many opportunities for future research, to better elucidate the mechanisms by which it acts in several metabolic and reproductive diseases. In the clinical settings, there are still numerous gaps to be filled before the role of irisin as a prognostic or diagnostic biomarker, or as a therapeutic target, can be established. The findings already published highlight that this molecule deserves further investigation.
